# Heritability of myopia and its relation with *GDJ2* and *RASGRF1* genes in Lithuania

**DOI:** 10.1186/s12886-018-0787-1

**Published:** 2018-05-24

**Authors:** Edita Kunceviciene, Margarita Sriubiene, Rasa Liutkeviciene, Ilona T. Miceikiene, Alina Smalinskiene

**Affiliations:** 1 Institute of Biology Systems and Genetic Research, Lithuanian University of Health Sciences, 18 Tilzes St, Kaunas, Lithuania; 2 Department of Ophthalmology, Lithuanian University of Health Sciences, 2 Eiveniu St, Kaunas, Lithuania

**Keywords:** *RASGRF1*, *GJD2*, Twin, Myopia

## Abstract

**Background:**

This study aimed to assess heritability of myopia in Lithuania and evaluate both genes *GJD2* (Gap Junction Protein, Delta 2) and *RASGRF1* (RAS protein-specific guanine nucleotide-releasing factor 1) relation with myopia.

**Methods:**

In this study Lithuanian twin population aged between 18 and 40 (*n* = 460) were examined. Single-nucleotide polymorphisms of the *RASGRF1* (rs8027411) and *GJD2* (rs634990) genes were assessed by real-time polymerase chain reaction method.

**Results:**

Intrapair correlations for spherical equivalent in all twin pairs were significantly higher in MZ twin pairs *r* = 0.539 (*p* < 0.001, 95% CI 0.353–0.684) than in DZ twin pairs *r* = 0.203 (*p* < 0.01, 95% CI 0.0633–0.442) in myopia group. Correlations for spherical equivalent in emmetropia group were not significant in MZ twin pairs *r* = 0.091 (*p* > 0.05, 95% CI -0.215-0.381) and in DZ twin pairs *r* = − 0.220 (p > 0.05, 95% CI -0.587-0.222). The odds ratio (95% CI) were 2.7 (1.018–7.460) for combinations of genotypes of rs634990 CC and rs8027411 GT (*p* = 0.046).

**Conclusions:**

Our studies have shown that the heritability of myopia makes 67.2% in Lithuania. Persons with combinations of genotypes rs634990 CC and rs8027411 GT have 2.7 times higher odds to have myopia.

## Background

Refractive error is one of the priority targets of World Health Organization Vision 2020 [1]. More than 150 million people in the world are estimated to be visually impaired because of uncorrected refractive error, 8 million of them are functionally blind [2]. Myopia affects approximately one-third of adults older than 20 years in the United States, and in areas with high prevalence, specifically in urban East Asia, more than 80% of students graduating from school are myopic [3]. It is estimated that by the year 2020, 2.5 billion people - one third of the world’s population will have been affected by myopia alone [4]. Worldwide, myopia affects approximately one in four individuals in western population and is the most common visual disorder [4, 5].

In Lithuania, the data on refractive errors are systematically collected, and the official statistics are published by the Lithuanian Department of Statistics. In the population of Lithuania from 2007 to 2014, the prevalence of myopia increased from 44.3 to 63.9 per 1000 population (www.sic.hi.lt).

Both genetic and environmental factors can affect development of myopia, but the exact causes are not fully understood [6]. The genetic contribution to a trait often is assessed through heritability. Heritability is the proportion of phenotypic variability in a population trait that is due to genetic differences [7]. Up to now, the heritability of myopia has not been studied in Lithuania.

Twin studies allow us to estimate the overall gene influence, and the results can show the heritability of myopia. The twin method assumes that monozygotic (MZ) twins are influenced by largely similar environmental differences as dizygotic (DZ) twins, but MZ twins share the same genes whereas DZ twins on average share only half their genes [8]. Twin method is excellent for the estimation of heritability of myopia, but it does not show the specific genes that may possibly be involved in the heritability of myopia. Thus, we chose genetic markers, in order to establish whether any of them are significantly associated with myopia.

Genome-wide association studies (GWAS) for refractive error showed that single nucleotide polymorphisms (SNPs) in 15q25 and 15q14 were associated with refractive error and myopia [9]. *RASGFR1* is a gene made of 28 exons. This gene has a significant influence on development of myopia. *RASGFR1* gene encodes Ras protein-specific guanine nucleotide-releasing factor-1, which is highly expressed in the retina and neurons. Then *RASGFR1* gene proceeds to activate Ras [10, 11]. Also, RASGRF1 is a nuclear exchange factor that promotes GDP/GTP exchange on the Ras family GTPases and is related to synaptic transmission of the photoreceptor responses [12]. Muscarinic receptors and retinoic acid can regulate *RASGRF1* expression as well [11]. Some animal and human studies showed that muscarinic inhibitors prevented the development of myopia [13]. In animal models of myopia there was detected reduced synthesis of choroidal retinoic acid [14]. To date, studies of SNP rs8027411 of *RASGRF1* gene associations with high myopia in different populations have provided controversial results [6, 15–17].

The *GJD2* gene at 15q14 encodes a neuron-specific protein connexin 36 (CX36), a 36 kDa protein, which is a neuron-specific protein of a family of integral membrane proteins [18]. CX36 forms gap junction channels between adjacent membranes of neuronal cells. It is present in photoreceptors, bipolar and amacrine cells, and, by enabling intercellular transport of small molecules and ions, plays an essential role in the transmission process of the retinal electric circuitry [18, 19].

The aim of our research was to find associations between the *GJD2*, *RASGRF1* genes and myopia development and to assess the heritability of myopia in Lithuania.

## Methods

### Ethics statement

Permission (Number P1–52/2005) to undertake the study was obtained from the Kaunas Regional Biomedical Research Ethics Committee. Before the study, the procedure and purpose of the study was explained, and an informed consent was obtained from all participants.

### Study samples

The twins participating in this study were from the Twin Centre of Lithuanian University of Health Sciences. The Twin Centre has registered more than 600 twin pairs who agreed to participate in various medical and genetic studies. The study was conducted in the Institute of Biological Systems and Genetics Research*,* Lithuanian University of Health Sciences.

### Refractive error measurement

Refractive error was measured with Sol. Cyclopentolate 1% using an autorefractor (Accuref-K9001, Shin-Nippon, Japan) and calculated by the mean spherical equivalent for each of the two eyes of every individual. The mean spherical equivalent was calculated using the standard formula: spherical equivalent = sphere+(cylinder/2).

MZ and DZ twins with spherical equivalent of at least one eye > = − 0.5 D were assigned to the myopia group. Twins whose spherical equivalent was between 0.49 and − 0.49 D were included in the emmetropia group. The myopia degree was determined by the strength or optical power of a corrective lens that focuses distant images on the retina: from − 0.5 D to − 3 D mild-degree myopia; from − 3 D to − 6.0 D medium-degree myopia; and − 6.0 D and over high-degree myopia [20, 21].

The exclusion criteria were as follows: 1) cataract, refractive surgery or other previous interventions that might have affected refractions; 2) other refractive errors; 3) refusal to participate in the research.

The inclusion criteria were as follows: 1) no ophthalmological eye disorders were found on detailed ophthalmological evaluation; 2) participation consent.

Lenses were evaluated by a slim-lamp biomicroscopy with the illumination source at a 45 degree angle and the light beam set being set to 2 mm width.

### Verification of zygosity

Zygosity was determined using a DNA test. The polymerase chain reaction set (AmpFlSTR® Identifiler®, Applied Biosystems, Foster City, CA, USA) was used to amplify short tandem repeats. 15 specific DNA markers were used for comparison of genetic profiles: D8S1179, D21S11, D7S820, CSF180, D3S1358, TH01, D13S317, D16S539, D2S1338, D19S433, vWA, TROX, D18S51, D5S818, and Amelogenin. The sample’s gender, age, zygosity characteristics and spherical equivalents are shown in Table 1.

**Table 1:** Characteristics of twin pairs, defined by zygosity

	MZ twins	DZ twins	*p* value
Sex, pairs			
Male	80	37	0.900^†^
Female	55	53	
Male/Female		5	
Total:	135	95	
Age, years			
Mean ± SE	24.10 ± 0.54	26.06 ± 0.93	0.379^‡^
Median	20.79	21.38	
Min, Max	18, 40	18, 40	
Spherical equivalent (D)			
OD Mean ± SE	−1.212 ± 0.102	−1.524 ± 0.196	0.667^‡^
Median	−0.75	−0.75	
Min, Max	−7.25, 0.375	−7.375, 0.49	
OS Mean ± SE	−1.112 ± 0.104	−1.449 ± 0.198	0.338^‡^
Median	−0.625	−0.75	
Min, Max	−7.25, 0.35	−6.75, 0.49	

*Abbreviations: MZ* – monozygotic twins*; DZ* - dizygotic twins, *D* diopters, *SE* Standard error, *p* > 0.05 – comparison between *MZ* and *DZ* twins

† *p* value for the chi-square test, ‡ *p* value for the Mann-Whitney test

### DNA extraction

Peripheral blood samples were collected from each individual in ethylenediaminetetraacetic (EDTA) tubes for DNA extraction. DNA was extracted from leukocytes using a reagent kit (NucleoSpin Blood L Kit; Macherey & Nagel, Düren, Germany). DNA samples from one member of each MZ pair were used for genotyping.

### Genotyping

SNP of the *GJD2* gene (rs634990) were assessed using a commercial genotyping kit C_2088259_10. SNP of the *RASGRF1* gene (rs8027411) was assessed using a commercial genotyping kit C_185318_10 (Applied Biosystems, Foster City, CA, USA). The Applied Biosystems 7900HT Real-Time Polymerase Chain Reaction System was used for detecting the SNPs. The cycling program started with heating for 10 min at 95 °C, followed by 40 cycles of 15 s at 95 °C and 1 min at 60 °C. Allelic discrimination was carried out using the software of Applied Biosystems. Both SNPs (rs8027411, rs634990) were present in two previous GWASs.

### Statistical analysis

The data was analysed with the statistical software package SPSS version 19.0 for Windows. Odds ratios and 95% confidence intervals were computed to assess the association between two SNPs and myopia multivariate logistic regression (Tables 3). Statistical significance was determined at a two-tailed *p* = 0.05 level. Estimate of heritability _(_h^2^) was obtained Pearson’s correlations (r) for MZ and DZ twin pairs: h^2^ = 2×(r_MZ_-r_DZ)_ [22].

## Results

230 pairs of twins (135 MZ and 95 DZ) aged between 18 and 40 participated in the study, their mean age being 25.08 years (SE 0.7 years) (Table 1). The mean spherical equivalent was − 1.324 ± 0.150, with a range from 0.49 D to − 7.375 D. There were no significant differences between MZ and DZ twins in age and spherical equivalent of the left and right eyes.

**Fig. 1:**
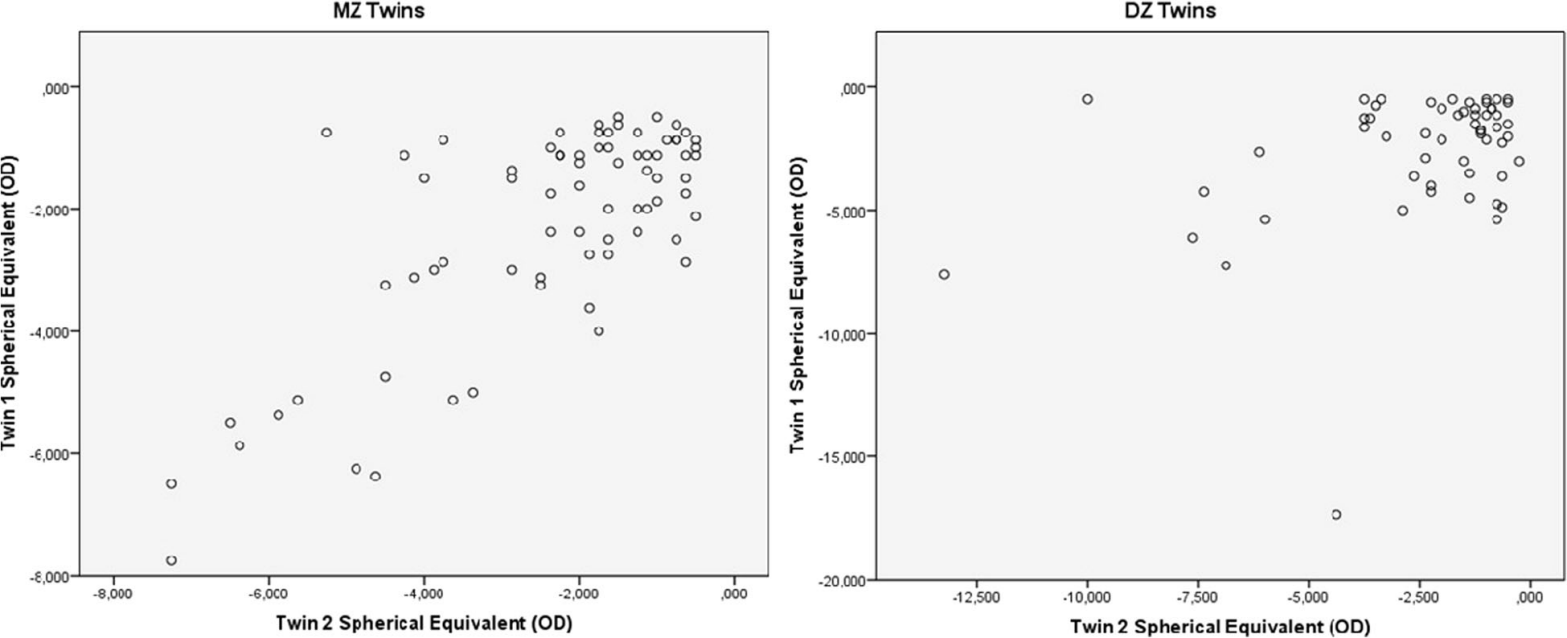
Scatter plots of refractive error as spherical equivalent for each twin‘s right eye, twin 1 versus twin 2 in 73 MZ and 56 DZ twin pairs in myopia group

**Fig. 2:**
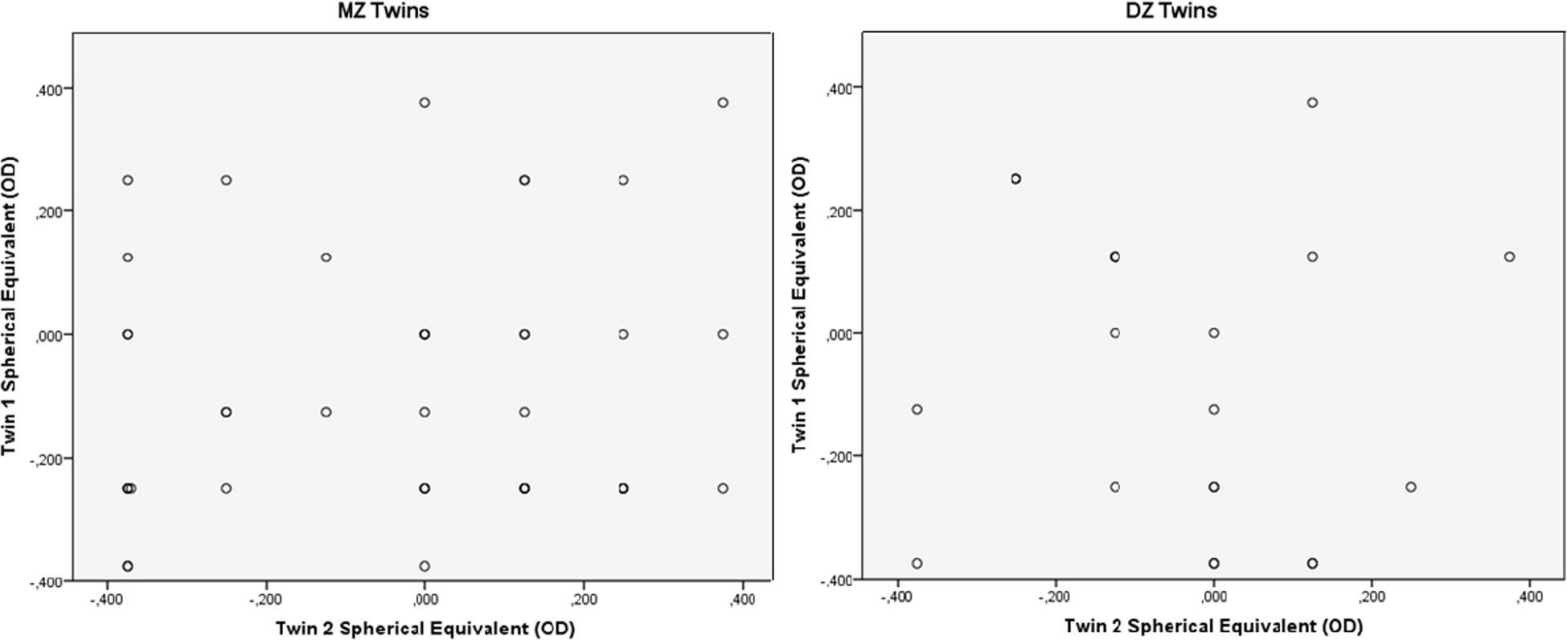
Scatter plots of refractive error as spherical equivalent for each twin‘s right eye, for twin 1 versus twin 2 in 65 MZ and 43 DZ twin pairs in emmetropia group

Refractive errors for twin 1 versus twin 2 for mean of spherical equivalent are shown in figs. 1 and 2. Intrapair correlations for spherical equivalent in twin pairs were significantly higher in MZ twin pairs *r* = 0.539 (*p* < 0.001, 95% CI 0.353–0.684) than in DZ twin pairs *r* = 0.203 (*p* < 0.01, 95% CI 0.0633–0.442) in myopia group. Correlations for spherical equivalent in emmetropia group were not significant: *r* = 0.091 (*p* > 0.05,995% CI -0.215-0.381) in MZ twin pairs and *r* = − 0.220 (p > 0.05, 95% CI -0.587-0.222) in DZ twin pairs. The correlations of MZ were clearly higher compared to DZ pairs, indicating genetic effects on myopia.

We genotyped two SNPs (rs634990 and rs8027411) in 189 myopia (83 MZ and 106 DZ) and 96 emmetropia (25 MZ and 71 DZ) subjects. The results are shown in Table 2. The distribution of the two SNP genotypes matched the Hardy-Weinberg equilibrium (*p* ≥ 0.05). High-degree myopia was present in 11 cases, 33 twins had medium-degree 10 and 145 twins mild-degree myopia. But we didn’t find significant correlations between myopia degree and genotypes.

**Table 2:** Frequency of *GJD2* and *RASGRF1* genotypes and allele (%)

SNP ID	Gene	N	Genotype Frequency			Minor allele frequency	P value
rs634990	*GJD2*	272	CC	CT	TT	C	0.1692
			56 (20.6%)	147 (54%)	69 (25.4%)	259 (47.6%)	
rs8027411	*RASGRF1*	285	GG	GT	TT	G	0.0913
			60 (21.1%)	157 (55.1%)	68 (23.8%)	277 (48.57%)	

5 models were used to calculate the odds ratios to have myopia separately with each gene (GJD2 or RASGRF1), results were not significant (Table 3, Table 4).

**Table 3:** Pooled results of the associations between *GJD2* and myopia

				95% C.I. For Exp(B)	
Model	Genotype	Sig.	Exp(B)	Lower	Upper
Codominant	CC CT TT	0.085 0.742 -	2.000 1.105 -	0.909 0.611 -	4.401 1.998 -
Dominant	(CC + CT) versus TT	0.390	1.284	0.727	2.268
Recessive	(CT + TT) versus CC	0.071	1.870	0.947	3.689
Overdominant	(CC + TT) versus CT	0.467	0.828	0.499	1.376
Additive	–	0.102	0.729	0.500	1.064

**Table 4:** Pooled results of the associations between *RASGRF1* and myopia

				95% C.I. For Exp(B)	
Model	Genotype	Sig.	Exp(B)	Lower	Upper
Codominant	GG GT TT	0.940 0.680 -	1.029 0.880 -	0.488 0.480 -	2.173 1.615 -
Dominant	(GG + GT) versus TT	0.774	0.918	0.513	1.645
Recessive	(GT + TT) versus GG	0.705	1.126	0.610	2.076
Overdominant	(GG + TT) versus GT	0.578	0.868	0.528	1.428
Additive	–	0.096	0.991	0.686	1.432

But we found significant association between the combinations of GJD2 CC and RASGRF1 GT and myopia (Table 5). The odds ratio of myopia compared to emmetropia (95% confidence intervals [CIs]) was 2.7 (1.018–7.460) for GJD2 CC and RASGRF1 GT genotypes.

**Table 5:** Odds ratio of myopia according to the combinations of *GJD2* and *RASGRF1*

Myopia					
Genotype				95% C.I. For Exp(B)	
*GJD2* *SNP ID rs634990*	*RASGRF1* *SNP ID rs8027411*	Sig.	Exp(B)	Lower	Upper
CC	GG	0.540	0.692	0.213	2.244
CC	GT	0.046†	2.756	1.018	7.460
CC	TT	0.335	1.898	0.516	6.980
CT	GG	0.460	1.407	0.569	3.482
CT	GT	0.284	0.747	0.439	1.273
CT	TT	0.706	0.864	0.405	1.845
TT	GG	0.990	1.006	0.391	2.587
TT	GT	0.129	0.542	0.246	1.195
TT	TT	0.857	1.096	0.403	2.986

†*p* < 0.05 comparison between myopia and emmetropia combinations of genotypes

The number with combinations of genotypes rs634990 CC and rs8027411 GT and myopia degrees are shown in Table 6.

**Table 6:** Distribution of SNP rs634990 and SNP rs8027411 genotypes according to degrees of myopia

Myopia degree	N	SNP ID rs634990 *N* = 272			SNP ID rs8027411 *N* = 285			Combinations of genotypes: SNP ID rs634990 + SNP ID rs8027411								
		CC	CT	TT	GG	GT	TT	CC + GG	CC + GT	CC + TT	CT + GG	CT + GT	CT + TT	TT + GG	TT + GT	TT + TT
High	11	4	5	2	3	6	2	1	2	1	2	2	1	0	2	0
Moderate	33	7	20	6	8	17	8	2	4	1	4	10	5	2	4	0
Low	145	32	80	20	34	83	28	8	18	6	18	50	12	9	10	1
Control	96	13	42	41	15	51	30	1	8	4	8	27	7	6	16	19

## Discussion

We estimated heritability of myopia according to correlations for MZ and DZ twin pairs and our study showed 67.2% heritability of myopia. Three published twin studies of refractive error have found high heritability from 84 to 86% [23], 89 to 94% [24] and 75 to 88% [25]. It is indicate that heritability in Lithuania is lower than in other Europian populations. Dirani et al. have reported that different populations have shown a wide range of heritability estimates ranging from 50 to 90% [25]. Results shown that the samples of the population and different methods may affect the estimates of heritability [25]. In our study 77% twins had mild-degree myopia, 17% - medium-degree and 6% - high-degree myopia. Meanwhile, the medium-degree myopia accounted for the largest portion in the mentioned studies of heritability in Europe.

Study showed that the gene *GJD2*, located nearest to the locus 15q14, and *RASGRF1* 15q25 are important for the transmission and processing of visual signals [23, 26]. The studies of genetic associations in some European and Japanese populations showed that common genetic variations located in *GJD2* and *RASGRF1* were associated with common myopia and refractive error [5, 11, 15].

A study of genome-wide associations (GWASs) showed associations of SNP with refractive error in 5328 individuals of the Dutch population which were not related. They found that carriers of the C allele of rs634990 have a higher risk of myopia [5]. Qiang et al. found that *RASGRF1* gene was significantly associated with high-degree myopia (risk allele T) but *GJD2* gene was not [15]. Also, results of meta-analysis, which included 2529 individuals with high-degree myopia and 3127 controls, showed that *RASGRF1* was significantly associated with high-degree myopia in Chinese and Japanese populations. However, carriers of the *RASGRF1 G* allele had a lower risk of high-degree myopia compared to carriers of the T allele (G versus T) [27]. Also, Hysi et al. found that individuals carrying TT alleles on the *RASGRF1* were significantly more likely to have myopia than those homozygous for the non-susceptibility GG alleles. We found a significant association between combinations of *GJD2* and *RASGRF1* genotypes and myopia. Our study showed that individuals with combinations of *GJD2* CC and *RASGRF1* GT genotypes were 2.7 times more likely to have myopia (*p* = 0.046). This indicates that some of our results are consistent with the previous reports. Individuals carrying CC alleles on the *GJD2* were significantly more likely to have myopia than carriers of TT alleles. But carriers of GT allele on the *RASGRF1* gene had more risk to have myopia than carriers of wild type alleles.

## Conclusion

Our studies have shown that the heritability of myopia makes 66.4% in Lithuania. We detected significant associations between the combinations of *GJD2* CC and *RASGRF1* GT and odds ratio of developing myopia.

## Abbreviations

D: Diopters
DZ: Dizygotic twins
GJD2: Gap Junction Protein Delta 2
MZ: Monozygotic twins
RASGRF1: RAS protein-specific guanine nucleotide-releasing factor 1
SE: Standard error
